# The Effects of Supplemental Feeding on Methane Emissions from Yak Grazing in the Warm Season

**DOI:** 10.3390/ani15040518

**Published:** 2025-02-12

**Authors:** Wanhao Ma, Muhammad Irfan Malik, Alan D. Iwaasa, Hong Wang, Hongli Wang, Jinfen Yang, Binqiang Bai, Jianwu Jing, Guangwei Hu, Lizhuang Hao, Shujie Liu

**Affiliations:** 1 Key Laboratory of Plateau Grazing Animal Nutrition and Feed Science of Qinghai Province, Qinghai University, Xining 810016, China; 2 Qinghai Yak Breeding Extension Service Center, Datong 810100, China; 3 Department of Veterinary Sciences, University of Turin, 10095 Turin, Italy; 4 Agriculture and Agri-Food Canada, Swift Current Research and Development Centre, Swift Current, SK S9H 3X2, Canada

**Keywords:** yaks, methane emissions, grazing, SF_6_ tracer

## Abstract

The yak industry constitutes a pivotal segment of livestock development across the Qinghai–Tibetan Plateau and adjacent pastoral regions. Existing studies have shown that supplemental feeding for grazing yaks significantly improves meat quality and growth performance, but its effect on enteric methane (CH_4_) emission from yaks has never been reported, so the present experiment aimed to investigate the effect of supplemental feeding of grazing yaks on CH_4_ emissions in three different periods of the warm season. Thirty male yaks were randomly assigned to two groups, with 15 yaks per group, The groups were designated as the grazing group (GR), with traditional grazing methods and with an initial body weight of 94.56 ± 3.9 kg, and the supplemental feeding group (GRS) with an initial body weight of 95.01 ± 4.1 kg, which received 1500 g/d of supplemental feed for 120 days. The methane emissions of the two groups were measured at the late regreening period (LRP), the greening-grass period (GGP), and the browning period (BRP) using the SF_6_ tracer method. The results showed that GRS increased (*p* < 0.01) the total DMI during warm season but decreased (*p* < 0.01) the forage intake at the LRP and GGP, resulting in a significantly reduced methane yield per kg of BW gain and per kg of DMD in warm-season grazing yaks (*p* < 0.01), a significantly reduced methane production per kg of DMI and methane energy to gross energy ratio in grazing yaks during the GGP (*p* < 0.05), and a similarly significantly decrease in methane production per kg of DMI and methane energy to gross energy ratio in grazing yaks during the BRP (*p* < 0.01). In conclusion, supplemental nutrition for grazing yaks during the warm season in alpine grasslands significantly enhances growth performance, reduces methane emissions, and improves dietary energy utilization efficiency.

## Simple Summary

Methane is the second most important greenhouse gas besides CO_2_; other than being a greenhouse gas, the production of methane from the rumen also causes a considerable loss of metabolizable energy. Yaks are the main livestock on the Tibetan Plateau and are well adapted to the extreme environment of the plateau. In a traditional production system, yaks graze on natural pastures throughout the year, but in recent years, with the increasing pressure on pastures, herders have also adopted the feeding method of grazing with supplemental concentrates for improving production. This study aimed to investigate the effects of supplemental feeding on methane emissions from grazing yaks during three different phenological periods in the warm season. The methane emission was evaluated using SF_6_ tracer technology. The results showed that during the warm season, the supplementation of a concentrate with alpine grasslands markedly lowered enteric methane emissions while enhancing dietary energy efficiency in grazing yaks.

## 1. Introduction

Methane emission results in the direct loss of metabolizable energy as well as contributes to the carbon footprint of yak production on the environment; the annual methane production from yaks in Qinghai Province is estimate to be 561,800 tons [1] annually; these calculations were based on the data and algorithm proposed by IPCC (2006) and combined with the daily methane production data of yaks grazing in the cold season measured by Ding et al. [2]. Currently, there are some studies on methane emission from yaks, but most of them focus on the in vitro simulation of rumen fermentation of forage and fodder in yaks [3] or the measurement of methane emission using respiratory calorimetry chambers [4,5]. The in vitro simulation technique reflects the degree of rumen fermentation to a certain extent, but its accuracy and precision are still not up to the demand of the research compared to the animal in vivo test. The in vivo measurement of methane emissions from yaks has been studied. Han et al. [6] used a closed-circuit respiratory mask calorimetric device to measure methane emissions from 90–110 kg to 140–160 kg yaks with different nutrient requirements, and the following equation was obtained using regression analysis: methane energy/total energy ingested (%) = 16.87 − 4.15 × multiples of maintenance requirements (r = 0.8175, *n* = 24). Ding et al. [2] used SF_6_ tracer technology to measure methane emissions from yaks during cold-season grazing and from yaks fully housed in alpine pastures and compared the magnitude of methane emissions between the two production methods. Currently, although a large body of evidence confirmed that yaks have evolved into domestic animals with efficient nutrient utilization [7,8], low methane emissions [2,9], and have adapted to the harsh natural environment of the plateau [10], 4.42% to 12.72% of the total energy of feeding is still lost to methane. Therefore, exploring further reduction in methane emission, improving energy utilization efficiency, and reducing the carbon footprint of the environment while regulating yak meat quality and improving production performance through nutrition will be one of the goals in yak production.

The present study investigates the effects of supplemental feeding on methane emissions from grazing yaks during three distinct periods of the warm season: the late regreening period (LRP), the greening-grass period (GGP), and the browning period (BRP), to test the following hypotheses: whether supplemental feeding reduces methane emissions per kg of dry matter intake in grazing yaks and whether concentrate supplementation improves energy utilization efficiency and growth performance in yaks. Methane emissions were measured using the SF6 tracer method during the LRP, GGP, and BRP. This study aims to fill the research gap regarding the impact of warm-season supplementation on methane emissions from grazing yaks and provide practical insights for improving yak production while reducing its environmental impact.

## 2. Materials and Methods

### 2.1. Location, Animals, and Diets

The experimental site is located in the hinterland of the Tibetan Plateau, Qinghai Province, Henan Mongolian Autonomous County of Qinghai Province, Saierlong Township alpine meadow pasture; the average plateau height is 3618 m; the average annual temperature is from −1.3 °C to 1.6 °C, while in the warm season, the temperatures reach ≥5 °C, with a maximum temperature of 13 ± 6 °C. The annual precipitation is 597~615 mm. The geographic latitude and longitude of the study area is from N 34°34′44.01″ to 34°35′21.68″ and from E 101°49′44.12″ to 101°50′47.06″. The major plant species in the pasture include *Kobresia humilis*, *Carex tristachya*, *Kobresia pygmaea*, *Potentilla fruticosa*, *Elymus nutan*, *Poa pratensis*, *Koeleria cristata*, *Saussureajaponica*, *Polygonum viviparum L*, *Scripustriqueter*, *Leguminosae*, *Pedicularis kansuensis*, *Ranunculus japonicus*, *Gentiana scabra*, *Gentiana macrophylla pall*, *Purple festuca*, and *Potentilla anserina*. Thirty healthy 2-year-old male yaks were selected, dewormed, and ear-marked. The 30 yaks were randomly divided into two groups, the grazing group (GR, *n* = 15, weighing 94.56 ± 3.9 kg) and the grazing with supplemental feeding group (GRS, *n* = 15, weighing 95.01 ± 4.1 kg). The sample size of 15 animals per group was guided by the practical limitations of conducting research in high-altitude alpine grasslands, particularly resource accessibility and logistical feasibility. This design aligns with established practices in similar experimental systems. The yaks were allowed to graze using the traditional grazing method on two separate pastures, and both pastures were connected with each other and had a uniform pasture and forage composition. The animals were shifted into their respective pastures at 7:30 a.m. and returned from the pastures at 19:30 p.m. to the paddocks. The GRS yaks received 750 g of pellet feed twice daily (pre- and post-grazing), totaling 1.5 kg/d. All the yaks were given free access to fresh and clean water in the pastures. The formulations and nutrient contents of the supplemental diets for the yaks are shown in Table 1. All yaks were weighed in the morning before shifting to the pastures at the end of each experimental period, with the weighing process occurring over two consecutive days.

The test period was divided into a pre-test period and a main test period. The three phases of the test, namely the late regreening period (LRP), the greening-grass period (GGP), and the browning period (BRP), were conducted from early June to early July, from mid-July to late August, and from early September to early October, respectively. The pre-test period of the digestibility test lasted for 15 days, and the main test period lasted for 7 days in each phenological phase. During the main test period, the fecal bags were applied to 10 yaks in each group, and the feces were collected for 7 consecutive days for each seasonal period at 4 h intervals, 6 times a day; the feces were weighed immediately. A representative sample (10%) of the 24 h collection period was stored at −20 °C in zipper bags. Forage samples were collected using a simulation method to follow the herd and observe the test yaks daily during the digestibility test period. The forage and fecal samples collected during the experimental period were analyzed for AIA content using the method described by Van Keulen and Young [11], and the amount of forage consumed by the yaks was calculated as follows:

I = F × F-AIA(%)/I-AIA(%), (1)

where I denote the amount of forage intake, F denotes the daily fecal excretion, and F-AIA and I-AIA denote the percentages of AIA in the fecal matter and forage, respectively.

The feed and fecal samples were analyzed for DM (105 °C) until constant mass, crude protein (CP) using the Kjeldahl method, Ca and P via AOAC [12], and acid detergent fiber (ADF) and neutral detergent fiber (NDF) using the Van Soest method [13]. Gross energy (GE) was measured with an adiabatic oxygen bomb calorimeter (6100; Parr Instrument Company, Moline, IL, USA), and the ether extract (EE) and the digestibility of dry matter (DMD) of supplementary concentrate were analyzed using the Soxhlet petroleum–ether extraction method and the two-stage technique [14], respectively.

### 2.2. Experimental Procedure for the Determination of Methane Emissions

Six yaks were selected from each group for the evaluation of enteric methane emission, and the SF_6_ osmosis tube with a stable release rate, as described later in the text, was inserted orally into the rumen; the rejection reaction of the test yaks to the osmosis tube was observed, and we reinserted it in time if the osmosis tube was spat out during rumination. After 3 d of observation, the test yaks can wear the gas collection device consisting of the head of the cage and the yoke-shaped PVC air collection tube which were not connected to the air circuit if they are normal. After acclimatization, the test yak entered the official period.

For each phase, the main test period for the evaluation of enteric methane emission is 10 d. On the first day of the test, the yoke, a yoke-shaped PVC air collection tube, was evacuated to a pressure of −70 kPa, and the pressure was checked on the following day, and a pressure of ±3 kPa was considered valid. Each morning at 08:00, prior to feeding, a yoke (−70 kPa) was placed around the neck of the test yak, and a special tie was used to secure the yoke and cage head; the quick connector was connected to collect the representative breath samples around the nostril of each yak for 24 h, while ambient air was collected for background values, and then the yoke was replaced with a new one. The final pressure of the removed yoke was measured to determine if it was necessary to increase the number of sample collections; a pressure of −50 kPa or so was considered an effective sample collection; otherwise, it was invalidated. The removed yoke was filled with high-purity nitrogen (99.999%) to a pressure of over 100 kPa and left for over 1 h to allow for the homogeneous mixing of the gases inside. A fine-tipped syringe was then used to rapidly withdraw the gas from the extraction port. It is essential that the syringe is thoroughly moistened and washed at least five times. The gas is then immediately extracted and injected into the gas collection bottle. The gas sample is stored at a low temperature and measured for as short a time as possible to avoid any alteration in the sample.

### 2.3. Gas Chromatographic Conditions for CH_4_ and SF_6_ Analyses

CH_4_ and SF_6_ were analyzed with a gas chromatograph (GC-2014, Shimadzu Corporation, Kyoto, Japan) under the following conditions: The chromatographic column utilized is a packed column Porapak. Column temperatures were adjusted to 80 °C (100 °C for SF_6_). A flame ionization detector (FID) is employed for the analysis of CH_4_, whereas an electron capture detector (ECD) is utilized for SF_6_. The temperature of the auxiliary (AUX) is maintained at 250 °C (300 °C for SF_6_). The injector temperature is set to 150 °C. The carrier gas tail pressure is adjusted to 0.2 MPa. The methane standard gas, with a concentration of 40.8 ppm, was obtained from Beijing Jinggao Gas Co., Ltd. (Beijing, China), while the SF_6_ standard gas, with a concentration of 99 ppt, was obtained from Beijing Hepu Gas Co., Ltd. (Beijing, China). A sample volume of 500 μL was injected manually, and single-point calibration was employed for the purposes of analysis and calculation.

### 2.4. Making SF_6_ Permeation Tubes and Measuring Permeation Rate

Large-sized permeation tubes and high-purity SF_6_ gas were selected to make SF_6_ permeation tubes, and a single permeation tube was filled with at least 2 g of SF_6_ gas, which was supplied by Alan Iwaasa’s group of the Semi-Arid Grassland Agricultural Research Centre, Department of Agriculture, Canada, and the purity of SF_6_ was 99.99%, which was purchased from Qinghai Xinhe Fluorine Industry Co. (Xining, China). The SF_6_ permeation tubes were incubated at 39 °C (simulating rumen temperature) in a desiccated chamber. Over a 3-month validation period, the tubes were weighed triweekly to confirm stable release rates. The tubes were made of chemically inert materials to prevent corrosion in the rumen, ensuring the stability of the permeation rate. The stable release rates were confirmed through regression equations, as detailed in Table 2. The high R^2^ values (ranging from 0.997 to 0.999) and the significant *p*-values (<0.001) indicate a consistent and reliable release rate throughout the study period.

### 2.5. Calculation of Methane Emissions

To ensure the accuracy of the data determined using the gas chromatograph instrument, each sample was injected at least six times until stable data values were obtained for the same sample. The CH_4_ emission was calculated on a daily basis according to the following formula [15]:

CH_4_Q = (CH_4_M − CH_4_B)/(SF_6_M − SF_6_B) × SF_6_Q × MW CH_4_/MW SF_6_, (2)

where CH_4_Q and SF_6_Q denote the daily emission of CH_4_ and the permeation rate of SF_6_ (g/d), respectively, CH_4_M and SF_6_M denote the concentrations of CH_4_ and SF_6_ measured in the yoke (μg/m^3^), CH_4_B and SF_6_B denote the concentrations of CH_4_ and SF_6_ measured in the background samples of the air (μg/m^3^), and MW CH_4_ and MW SF_6_ denote the molecular weights of CH_4_ and SF_6_.

### 2.6. Statistical Analysis

The experimental data were analyzed using One-way ANOVA in SPSS v26.0 (IBM SPSS Statistics, SPSS Inc., Chicago, IL, USA). The normality of the data distribution was confirmed using the Shapiro–Wilk test (*p* > 0.05 for all groups). The homogeneity of variances was assessed via Levene’s test; if violated (*p* < 0.05), Welch’s ANOVA was employed to address unequal variances. Means comparisons were carried out using the least significant difference (LSD) method, with a significance level of *p =* 0.05. The standard error of the mean (SEM) was used to represent the variability of the means.

## 3. Results

### 3.1. Dry Matter Intake and Growth Performance

Supplemental feeding during the warm season significantly enhanced yak growth performance across all phenological periods (Table 3). The GRS exhibited higher final body weight (FBW) and ADG compared to the GR (*p* < 0.01), with the most pronounced improvement observed during the BRP. Specifically, the GRS achieved 1.57 to 2.70 times greater ADG than the GR across all phenological periods (*p* < 0.01), with peak improvements observed during the BRP, highlighting the compensatory growth potential of yaks under nutritional supplementation. DMI in GRS yaks increased during LRP, GGP, and BRP compared to GR (*p* < 0.01), while forage intake decreased in GRS during LRP and GGP (*p* < 0.01).

### 3.2. Methane Emissions Across Warm Season

Supplemental feeding consistently reduced methane emission intensity, with notable variations across phenological periods (Table 4, Table 5 and Table 6).

LRP: significant decreases occurred in methane yield per kg body weight (Ym/BW) and dry matter digested (Ym/DMD) (*p* < 0.01).

**Table 4:** Effect of supplementary feeding on methane emission from yaks grazing on alpine grasslands in LRP.

Items	GR	GRS	SEM	*p*-Value
Ym/DMI (g/kg)	16.58	14.55	0.630	0.109
Ym/BW (g/kg)	147.59 ^a^	99.93 ^b^	6.902	<0.001
Ym (g/day)	71.45	74.17	2.824	0.639
Ym/DMD (g/kg)	23.02 ^a^	18.02 ^b^	0.981	0.008
Ym/BW^0.75^ (g/d∙kg^0.75^)	2.05	1.98	0.077	0.662
CH_4_-E/GE (%)	4.86	4.30	0.189	0.144

Different superscripts on means in a row indicate a significant difference. Ym, methane yield; DMI, dry matter intake; BW, body weight; DMD, dry matter digested; CH_4_-E, methane energy; GE, gross energy.

GGP: Marked reductions were observed in Ym/DMI (*p* < 0.05), Ym/BW (*p* < 0.01), and Ym/DMD (*p* < 0.01). The CH₄-E/GE ratio also decreased (*p* < 0.05), suggesting improved energy utilization efficiency concurrent with elevated total dry matter consumption.

**Table 5:** Effect of supplementary feeding on methane emission from yaks grazing on alpine grasslands in GGP.

Items	GR	GRS	SEM	*p*-Value
Ym/DMI (g/kg)	18.13 ^a^	15.11 ^b^	0.718	0.033
Ym/BW (g/kg)	193.15 ^a^	114.26 ^b^	10.372	<0.001
Ym (g/day)	72.59	78.52	3.153	0.356
Ym/DMD (g/kg)	27.84 ^a^	19.26 ^b^	1.261	<0.001
Ym/BW^0.75^ (g/d∙kg^0.75^)	1.90	1.80	0.077	0.531
CH_4_-E/GE (%)	5.43 ^a^	4.54 ^b^	0.226	0.045

Different superscripts on means in a row indicate a significant difference. Ym, methane yield; DMI, dry matter intake; BW, body weight; DMD, dry matter digested; CH_4_-E, methane energy; GE, gross energy.

BRP: This period exhibited the most pronounced mitigation effects, with substantial declines in Ym/DMI, Ym/BW, and Ym/DMD (*p* < 0.01). A significant reduction in CH₄-E/GE was also recorded (*p* < 0.01), demonstrating enhanced metabolic efficiency during this phase.

**Table 6:** Effect of supplementary feeding on methane emission from yaks grazing on alpine grasslands in BRP.

Items	GR	GRS	SEM	*p*-Value
Ym/DMI (g/kg)	23.89 ^a^	18.93 ^b^	0.883	0.003
Ym/BW (g/kg)	395.50 ^a^	176.26 ^b^	24.438	<0.001
Ym (g/day)	77.88	87.68	3.682	0.188
Ym/DMD (g/kg)	41.23 ^a^	26.02 ^b^	1.771	<0.001
Ym/BW^0.75^ (g/d∙kg^0.75^)	1.95	1.82	0.077	0.426
CH_4_-E/GE (%)	7.13 ^a^	5.68 ^b^	0.277	0.007

Different superscripts on means in a row indicate a significant difference. Ym, methane yield; DMI, dry matter intake; BW, body weight; DMD, dry matter digested; CH_4_-E, methane energy; GE, gross energy.

### 3.3. Differences in Methane Emissions Between Cattle and Yaks

Yaks demonstrated distinctively lower methane emissions compared to grazing cattle (Table 7). Daily methane production in yaks (71.4–87.7 g/day) was 60–80% lower than dairy cows (228–431 g/day) and 30–50% lower than beef cattle (70–237 g/day). Methane yield per kg DMI (14.6–23.9 g/kg) and metabolic body weight (1.82–2.05 g/d·kg^0.75^) were also significantly reduced (*p* < 0.01).

The integration of the findings of previous research with the data obtained in the present study reveals that yaks exhibit comparatively lower levels of daily methane emissions, methane yield per kg of DMI, methane yield per kg of metabolic body weight, and the ratio of methane energy to gross energy in comparison to grazing dairy and beef cattle.

## 4. Discussion

### 4.1. Effect of Supplementary Feeding on Growth Performance in Yak Grazing in Warm Seasons

The intake in grazing livestock is influenced by numerous factors, and its regulatory mechanisms are complex [38,39]. The forage intake is affected by the physical characteristics of the diet, rumen filling effects, and the nutrient concentration of the feeding [39]. Supplemental feeding for grazing ruminants reduces forage intake by decreasing grazing time [40]. The results of this study revealed that concentrate supplementation during the warm season effectively reduced the intake of alpine grass forage by grazing yaks, while their total intake was higher. The decrease in forage intake in the GRS could be associated with the nutrient composition of the supplemented diet, which was more digestible and easily fermented in the rumen, increasing the rate of rumen chyme flow and further increasing total feed intake [29].

The nutrient availability of forage in alpine grasslands varies greatly throughout the year, especially during the cold season when the quality and quantity of forage are extremely low for 7–8 months; grazing yaks are severely deprived of forages, as they are unable to meet their nutritional requirements. It has been shown that yaks lose about 25% of their body weight after the entire cold season [41], which is a serious loss. Studies have shown that the supplemental feeding of grazing yaks in the cold season can significantly increase growth performance and enhance overwintering ability [42]. In the current experiment, the ADG of GRS yaks were 1.57, 1.86, and 2.70 times higher than that of grazing yaks at the LRP, GGP, and BRP, respectively. Supplementary feeding in the warm season significantly improved growth performance. It can be seen that with the delay of the phenological period and the decline of pasture quality, the effect of supplementation on the growth performance of yaks was greater, and the poorer the quality of grass pasture, the more prominent the effect of nutritional supplementation on yak growth. This trend is attributable to compensatory growth mechanisms exhibited by grazing yaks, as well as the high quality and abundance of warm-season forage availability. Additionally, the supplemental feeding of concentrates also promotes the yaks to fulfill their growth potential, enabling high growth performance during the warm grazing season. The study showed that after enduring a lengthy and harsh cold season, the yaks that continued to graze during the subsequent warm season exhibited a significant increase in their ADG, reaching 419.33 g/d. In contrast, the control group of yaks fed indoors throughout the trial recorded an ADG of 247.47 g/d, clearly demonstrating the property of compensatory growth in yaks against the nutritional stress of the cold season [43]. Our finding suggested that the supplementation of concentrate during the warm season in alpine meadows could be a good strategy to improve the growth performance and attain the maxim potential from grazing yaks.

### 4.2. Effect of Supplementary Feeding on Methane Emissions from Yak Grazing in Warm Seasons

Several studies have confirmed that yaks have adapted to the harsh environment of nutrient deprivation in alpine pastures, as evidenced by research on yak tongue histomorphology [44], behavior [45,46,47], energy and protein utilization [7,8,48], methane emission [2,9], and rumen microbes [9,10]. Currently, yak production in Tibetan areas suffers from the problem of low production performance and low profit. With the economic and social development in Tibetan areas, there is an urgent need to increase the income of herdsmen. To address the bottlenecks that require prompt solutions in production and achieve efficient yak production as well as high earnings for herdsmen, the appropriate supplementary feeding of grazing yaks has become the optimal choice at present. When labor costs are excluded from the calculation, the GR demonstrates virtually zero input, whereas the GRS requires only a daily input of 1.5 kg of concentrate feed. Based on the concentrate feed procurement price of 3.4 CNY/kg and the market price of yak meat at 56 CNY/kg, net income comparisons derived from ADG data reveal that the GRS generated an additional daily income of 10.3 CNY, 13.3 CNY, and 13.9 CNY compared to the GR during the LRP, GGP, and BRP, respectively. When aggregated across the entire warm season, the composite advantage reached 12.5 CNY per day. These results indicate that moderate concentrate supplementation during the warm season can substantially enhance economic returns for herders. The supplementary feed utilized in this study was formulated based on previous research findings by our team on the nutritional value assessment of forage in warm-season pastures. The supplementary feed contained 12% of heat-treated rapeseed. Through the analysis of feed intake, it was calculated that the dietary fat content consumed by yaks at the late regreening stage was 4.75%. In comparison, the forage fat content for the grazing group was 3.09%, indicating that the supplementary feeding group had a 1.66% higher fat content than the grazing group. Grainger and Beauchemin [49] conducted a meta-analysis of numerous studies on the impact of adding fat to ruminant diets on methane emissions. The results indicated that when the dietary fat content was below 8%, an increase of 1% in dietary fat content could reduce methane emissions by 1 g/kg per kg of DMI, regardless of the composition, source, and form of addition (oil or seed). Another study showed that a 1% increase in dietary fat content reduced methane emissions by 0.79 g CH_4_/kg DMI [50]. The spraying of canola oil on forage grasses on methane emissions from grazing cattle indicated that for every 1% increase in fat content, methane emissions decreased by 0.8 g CH_4_/kg DMI [29]. In the current experiment, fat content was increased from 3.09% in the grass of the pasture to 4.75% in the supplemented group, which resulted in a 1.66% increase in fat and a decrease in methane emission of 2.03 g/kg DMI, and calculations showed that a 1% increase in fat content in this experiment resulted in a decrease in methane emission (1.22 g CH_4_/kg DMI).

### 4.3. Effect of Supplementary Feeding on Methane Emission from Yaks Grazing on Alpine Grassland in LRP

The nutrient availability in the pasture at the LRP of the alpine grassland is the highest throughout the year with the highest CP and the lowest NDF and ADF content [51], and therefore, the methane emission per kg dry matter consumed by grazing yaks is the lowest of the whole year during this period. Numerous warm-season supplemental feeding trials have confirmed that the energy, protein, and mineral nutrients provided by warm-season alpine natural pasture do not meet the nutritional requirements for efficient yak production [52,53,54], so supplemental feeding has become one of the most effective means to improve the productivity of warm-season grazed yaks and to reduce methane emissions. The supplementary feeding of grazing yaks extremely reduced methane emissions per kg BW gain and per kg DMD but had no effect on methane emissions per kg DMI and methane yield based on metabolic body weight. This could be associated with the high nutritive value of the pasture and the best weight gain of grazing yaks in this period. The decrease in methane emissions and abatement measures by improving roughage quality, digestibility, and dietary intake is the preferred choice for methane mitigating strategies [55,56,57]; additionally, improving ruminant growth performance is central to reducing the carbon footprint of livestock products [58], which corroborates with the results of higher DMI and ADG and lower per kg BW gain in the GRS of present study. Therefore, supplemental feeding in the LRP can significantly improve the weight gain performance of grazing yaks while reducing the carbon footprint of yak products. At the same time, in the late greening period, the grazing yaks were in the stage of compensatory growth, which ended the nutrient deprivation period of more than half a year, and the higher nutrient level of supplemental feeding for the grazing yaks would help them make full use of the characteristics of compensatory growth to increase the body weight gain [53]. By reducing methane emissions per kg body weight gain, the loss of metabolizable energy from methane emission can be reduced, and the utilization of dietary energy can be improved via supplemental feeding [2,6].

### 4.4. Effect of Supplementary Feeding on Methane Emission from Yaks Grazing on Alpine Grasslands in GGP

Due to the fact that pasture grasses produce the highest amount of grass during the GGP, the nutrient output per unit area of grass is the highest during this period, and although this provides sufficient forage for grazing yaks, the nutrient supply is still not enough to satisfy the nutrients required for yaks to reach their maximum growth potential, which is similar to the case of grasses at the end of the greening period, and therefore, the continuation of supplemental feeding will be the best option to enhance the performance of yaks during the whole warm season. The research conducted by Du et al. [51] indicates that the ether extract of forage during the GGP of the alpine grassland is significantly higher than that during the regreening period. Moreover, the conventional nutrient outputs such as crude protein, ether extract, and neutral detergent fiber during the GGP are more than twice as high as those during the regreening period. Consequently, the dietary nutrient level and total energy content consumed by grazing yaks during the GGP are greatly enhanced, leading to significant reductions in multiple indicators, including methane emissions per kg DMI, per kg DMD, or per kg BW gain, in the supplementary feeding group compared to the grazing group.

### 4.5. Effect of Supplementary Feeding on Methane Emission from Yaks Grazing on Alpine Grasslands in BRP

The grazing yaks during the BRP had the highest methane production among the three periods, both in the grazing and supplemental feeding groups, which is also in agreement with the previous findings [55,56,57,58,59] that the dietary nutritional level is one of the main factors determining the level of methane emissions from ruminants. By comparing the fat content of the diets of the grazing and supplemental feeding groups, it can be seen that the fat content of the diets of the supplemental feeding group increased by 1.77% compared to that of the grazing group, whereas NDF and ADF decreased by 11.58% and 11.49%, respectively, which was a greater decrease than that of the other two periods, which showed that an increase in the fat content and a decrease in the fiber content of the diet can substantially reduce methane emissions, and lower fiber content could substantially reduce methane emissions from ruminant livestock. During the BRP, when natural forage was characterized by elevated fiber content (NDF/ADF) and reduced digestibility, the supplemental diet (with higher fat and lower fiber) not only compensated for energy deficits but also redirected rumen fermentation pathways. This shift reduced hydrogen availability for methanogenesis while promoting propionate synthesis, a phenomenon consistent with studies on lipid supplementation in ruminants [49,50]. The nutritional status of natural forage, which varies with phenological changes, ultimately leads to significant changes in the structure of the rumen microbiota in yaks [60]. However, the microbiota quickly stabilizes to better utilize the nutritional components of the forage [61]. As mentioned earlier, the addition of fat to the diet is not incompatible with increased nutritional levels. In the present study, the supplementary feed was supplemented with heat-treated rapeseed, which contains about 21% protein and up to 46% fat, in addition to high levels of unsaturated fatty acids [62], making it a better source of protein and polyunsaturated fatty acids for livestock. For a long time, the use of rapeseed as a source of feed ingredients for livestock has been affected by its own glucosinolate and erucic acid content, and these anti-nutritional factors have severely limited its use in animal feed [62]. However, there are some reports that the heat treatment of rapeseed significantly reduces toxic glycosides, thioglucosides, and erucic acid content [63,64]. In addition to processing to reduce anti-nutritional factors, in recent years, plant breeders have developed a series of double-low oilseed rape varieties with low levels of erucic acid and glucosinolates through different breeding methods, which significantly reduce the content of anti-nutritional factors, providing the basis for a wide range of applications in livestock production [65]. Some scholars have shown that the addition of heat-treated rapeseed to housed yak diets can significantly increase yak growth performance and tenderness, improve yak meat quality and beneficial fatty acid content [66], as well as significantly reduce methane emissions [67], and all of these studies provide useful references for the present study. The supplemental feeding of concentrate containing heat-treated rapeseed to yaks grazing in the late greening, green-grass, and BRPs of the warm season can significantly reduce methane production and improve the efficiency of energy utilization of the diet, which can provide technical support for the efficient production of yaks grazing in the warm season.

### 4.6. Comparison the Differences in Methane Emissions Between Cattle and Yaks

The SF_6_ tracer technique has emerged as a widely adopted method for quantifying methane emissions from grazing ruminants, with continuous refinements enhancing its precision [68]. Comparative analyses using this technique reveal that yaks exhibit substantially lower daily methane output, reduced methane yield per kg of DMI, and decrease emissions per kg of metabolic body weight compared to cattle breeds (Table 7). These disparities likely stem from the yak’s unique evolutionary adaptations to the Tibetan Plateau’s extreme conditions. Notably, yaks possess a smaller body size and lower basal metabolic rate than cattle, which correlates with decreased energy demands and methanogenesis [69].

Yak rumen microbiomes demonstrate greater taxonomic diversity and lower methanogen abundance (e.g., *Methanobrevibacter* spp.) compared to cattle [10]. This community structure favors energy-efficient pathways, as evidenced by in vitro studies showing 20–30% less methane production from yak rumen fluid under identical substrate conditions [9]. Enhanced propionate synthesis via the succinate pathway in yaks redirects hydrogen away from methanogenesis, while accelerated digesta passage rates limit substrate availability for methane-producing archaea [10,70]. The abundance of hydrogen-producing microorganisms, hydrogen-consuming microorganisms, and methanogenic hydrogen-utilizing microorganisms in the rumen of yaks is different from that in cattle, and the difference in total hydrogen production in the rumen leads to the variation in methane production [71]. The results of metagenomic analysis indicate that the rumen of yaks has unique methanogenic pathways, resulting in lower methane production [72]. While increased DMI generally elevates methane emissions in ruminants [73], yaks maintain lower overall output despite compensatory intake adjustments. This is partly attributed to their natural diet in alpine grasslands, which includes lipid-rich forages that inhibit methanogen activity [74,75,76]. Collectively, these anatomical, microbial, and metabolic adaptations, including upregulated VFA production, reduced maintenance energy requirements, and efficient nutrient absorption, enable yaks to achieve superior energy utilization efficiency with minimal methane loss [77,78,79,80].

## 5. Conclusions

The supplementary feeding of yaks through warm-season grazing in an alpine pasture resulted in significant reductions in methane emissions per kg of DMI, methane emissions per kg of body weight gained, and methane energy to gross energy ratios in all three periods, as well as improved the efficiency of dietary energy use.

## Figures and Tables

**Table 1:** Supplementary concentrate diet formulation and its nutrient contents (DM basis).

**Ingredient Composition**	**Content (%)**	
Corn	44.90	
Wheat bran	25.25	
Double low rapeseed meal	12.65	
Heat-treated rapeseed	12.00	
Stone powder	2.50	
Premix ^1^	1.00	
Bentonite	1.00	
Salt	0.60	
CaHPO_4_	0.10	
**Nutritional Composition (** **DM** **basis), % Unless Mentioned**		
DM	93.61	
CP	18.15	
EE	8.76	
NDF	20.5	
ADF	8.25	
Ca	1.21	
P	0.79	
NaCl	0.66	
GE (MJ/kg)	18.38	
DMD	86.71	
ME (MJ/kg) ^2^	13.07	

^1^ Premix supplied per kilogram of dietary DM: vitamin A 1000 IU, vitamin D3 300 IU, vitamin E 1500 IU, biotin 1000 μg, phyllotaxy 10 mg, nicotinic acid 3000 mg, Fe 3600 mg, Cu 800 mg, Zn 3800 mg, Mn 3200 mg, KI 90 mg, Se 70 mg, and plumber ≤ 10. ^2^ MEs were calculated values, and others were measured values (ME = GE × DMD% × 0.82).

**Table 2:** Regression equation of the SF_6_ permeation rate.

Permeation Tubes	Regression Equation	R^2^	*p*-Value
1	y = −0.006x + 47.683	0.999	<0.001
2	y = −0.006x + 48.111	0.998	<0.001
3	y = −0.005x + 49.021	0.998	<0.001
4	y = −0.005x + 49.155	0.997	<0.001
5	y = −0.006x + 49.667	0.999	<0.001
6	y = −0.006x + 47.897	0.999	<0.001
7	y = −0.006x + 48.607	0.999	<0.001
8	y = −0.005x + 48.977	0.999	<0.001
9	y = −0.007x + 49.491	0.998	<0.001
10	y = −0.006x + 48.879	0.999	<0.001
11	y = −0.006x + 48.272	0.999	<0.001
12	y = −0.005x + 50.002	0.998	<0.001

**Table 3:** Effect of yak supplementary feeding on the production performance of grazing yaks in alpine grasslands in the warm season.

Items		GR	GRS	SEM	*p*-Value
IBW (kg)		94.56	95.01	0.719	0.762
FBW (kg)	LRP	113.98 ^a^	125.44 ^b^	1.375	<0.001
	GGP	129.17 ^a^	153.77 ^b^	2.505	<0.001
	BRP	137.15 ^a^	175.33 ^b^	3.691	<0.001
FDMI (kg/d)	LRP	4.31 ^a^	3.62 ^b^	0.081	<0.001
	GGP	4.01 ^a^	3.69 ^b^	0.056	0.003
	BRP	3.27	3.13	0.063	0.299
SDMI (kg/d)	LRP	0.00	1.50	—	—
	GGP	0.00	1.50	—	—
	BRP	0.00	1.50	—	—
DMI (kg/d)	LRP	4.31 ^a^	5.12 ^b^	0.090	<0.001
	GGP	4.01 ^a^	5.19 ^b^	0.119	<0.001
	BRP	3.27 ^a^	4.63 ^b^	0.141	<0.001
ADG (g/d)	LRP (40 d)	486 ^a^	761 ^b^	29.219	<0.001
	GGP (40 d)	380 ^a^	708 ^b^	35.772	<0.001
	BRP (40 d)	200 ^a^	539 ^b^	36.460	<0.001
	Warm seasons (120 d)	354.95 ^a^	669.39 ^b^	30.340	<0.001

Different superscripts on mean in a row indicate a significant difference. IBW, initial body weight; FBW, final body weight; FDMI, dry matter intake of forage; SDMI, dry matter intake of concentrate supplements; DMI, total dry matter intake of forage and concentrate supplements; ADG, average daily gain.

**Table 7:** Comparative methane emission metrics between yaks and cattle measured via SF_6_ tracer technology.

Source	Animal	Feeding Method	BW (kg)	DMI (kg)	Ym (g/day)	Ym/DMI (g/kg)	Ym/BW^0.75^ (g/d∙kg^0.75^)	CH_4_-E/GE (%)
Maciel et al. [16]	Beef cattle	Grazing	242.50	6.23	98.05	16.76	1.60	-
Neto et al. [17]	Beef cattle	Grazing + concentrate (high starch source)	259.11	7.69	127.63	17.14	1.98	4.38
	Beef cattle	Grazing + concentrate (high starch source with oil)	239.45	7.70	117.74	15.36	1.93	3.37
	Beef cattle	Grazing + concentrate (low starch source with oil)	257.55	7.45	114.61	15.45	1.78	3.39
	Beef cattle	Grazing + concentrate (low starch source)	246.66	7.85	120.48	15.44	1.94	3.49
Neto et al. [18]	Beef cattle	Grazing + concentrate (high starch source)	428.00	12.80	145.00	11.30	1.54	2.65
	Beef cattle	Grazing + concentrate (high starch source with oil)	409.00	12.70	115.00	9.07	1.26	2.04
	Beef cattle	Grazing + concentrate (low starch source with oil)	405.00	12.60	116.00	9.25	1.28	2.10
	Beef cattle	Grazing + concentrate (low starch source)	412.00	12.60	140.00	11.00	1.53	2.63
Richmond et al. [19]	Beef cattle	Grazing (lowland)	407.00	8.68	176.00	20.70	1.94	6.02
	Beef cattle	Grazing (upland)	407.00	9.55	202.00	21.60	2.23	6.48
Boland et al. [20]	Beef heifers	Grazing (high-quality pasture)	346.00	6.44	122.00	21.10	1.52	6.10
	Beef heifers	Grazing (low-quality pasture)	346.00	6.50	120.00	19.30	1.50	5.60
Chaves et al. [21]	Beef heifers	Grazing (different pasture quality)	379.00	5.40 to 6.50	144.00 to 158.00	22.20 to 29.20	1.68 to 1.82	6.90 to 9.60
Dini et al. [22]	Beef heifers	Grazing (high-quality pasture)	363.09	10.10	160.00	21.60	1.92	7.00
	Beef heifers	Grazing (low-quality pasture)	363.09	5.50	109.00	23.60	1.31	7.90
Hammond et al. [23]	Beef heifers	Grazing	230.00	8.69	202.00	23.00	3.42	-
Silveira et al. [24]	Beef heifers	Grazing	256.00	-	189.00	-	2.95	-
Andrade et al. [25]	Steer	Grazing (different pasture type)	213.00	6.70 to 7.80	146.00 to 180.00	22.90 to 25.30	2.60 to 3.20	-
Boadi et al. [26]	Steer	Grazing	342.80	11.10	222.63	20.06	2.79	6.70
	Steer	Grazing + barley grain	346.50	12.90	237.47	18.41	2.96	6.40
Carvalho et al. [27]	Steer	Grazing + concentrate	440.00	11.50	114.00	9.51	1.12	3.39
	Steer	Grazing + concentrate (supplemented linseed oil)	443.00	11.00	70.20	7.26	0.69	2.48
	Steer	Grazing + concentrate (supplemented palm oil)	430.00	11.60	112.00	9.91	1.08	3.36
	Steer	Grazing + concentrate (supplemented protected fat)	450.00	12.00	101.00	8.74	0.98	2.89
	Steer	Grazing + concentrate (supplemented soybean)	444.00	12.80	82.40	6.61	0.84	2.27
Fraser et al. [28]	Steer	Grazing (lowland)	386.00	10.30	216.00	21.00	2.48	6.70
	Steer	Grazing (upland)	410.00	7.70	173.00	22.90	1.90	6.80
Pinares-Patiño et al. [29]	Steer	Grazing + oil spray	293.80	8.29	138.60	17.20	1.95	4.96
	Steer	Grazing	293.70	7.51	156.10	20.90	2.20	6.28
de Souza et al. [30]	Steer	Grazing (different intensities)	237.00	5.98 to 7.31	171.00 to 227.00	25.20 to 30.60	2.30 to 2.86	-
McCaughey et al. [31]	Steer	Grazing	397.83	13.82	195.88	14.17	2.20	4.50
e Silva et al. [32]	Dairy cow	Grazing + concentrate	524.00	15.40	228.30	13.90	2.10	-
	Dairy cow	Grazing + concentrate (sunflower oil)	524.00	15.40	291.00	18.00	2.70	-
Lassey et al. [33]	Dairy cow	Grazing	483.00	12.88	262.80	20.40	2.55	6.16
Muñoz et al. [34]	Dairy cow	Grazing + concentrate (1kg/d)	494.00	17.00	323.00	19.40	3.08	6.40
	Dairy cow	Grazing + concentrate (5kg/d)	494.00	18.90	357.00	19.00	3.41	6.40
Ulyatt et al. [35]	Dairy cow	Grazing	475.00	19.30	431.00	22.33	4.24	6.80
Ulyatt et al. [36]	Dairy cow	Grazing	438.00	15.60	363.00	23.27	3.79	7.10
McCaughey et al. [37]	Lactating beef cow	Grazing	516.20	9.70	294.69	30.38	2.72	9.50
Ding et al. [2]	Yak	Grazing	174.00	3.78	81.40	21.50	1.70	7.30

## Data Availability

The corresponding author may provide supporting data for this research upon reasonable demand.
